# COVID-19 Pandemic Worsening Gender Inequalities for Women and Girls in Sub-Saharan Africa

**DOI:** 10.3389/fgwh.2021.686984

**Published:** 2021-07-29

**Authors:** Bright Opoku Ahinkorah, John Elvis Hagan, Edward Kwabena Ameyaw, Abdul-Aziz Seidu, Thomas Schack

**Affiliations:** ^1^Faculty of Health, School of Public Health, University of Technology Sydney, Sydney, NSW, Australia; ^2^Department of Health, Physical Education, and Recreation, University of Cape Coast, Cape Coast, Ghana; ^3^Neurocognition and Action-Biomechanics-Research Group, Faculty of Psychology and Sport Sciences, Bielefeld University, Bielefeld, Germany; ^4^L & E Research Consult, Wa, Ghana; ^5^Department of Population and Health, University of Cape Coast, Cape Coast, Ghana; ^6^College of Public Health, Medical and Veterinary Services, James Cook University, Townsville, QLD, Australia

**Keywords:** COVID-19, girls, inequity, sub-Saharan Africa, women

## Abstract

Pandemics such as COVID-19 have often resulted in international, national and sub-regional crises, with considerable inequities across many societies. With the already existing structural and socio-economic inequalities in sub-Saharan Africa, the stay-at-home orders, lockdowns, and shutdowns across the sub-regional states could worsen and have a tremendous impact on vulnerable groups. Numerous studies across a variety of contexts have well-documented gender, and cultures on varied health outcomes. However, these have not been contextualized in sub-Saharan Africa in the light of the COVID-19 pandemic. This mini review discusses the ways by which COVID-19 has impacted the lives of girls and women across sub-Saharan Africa and the strategies that can help mitigate these challenges. The mini review specifically shares light on a wide array of dimensions where the inequities exist. These include the disproportionate areas affected by COVID-19; household inequities; educational inequalities; work/employment inequalities; disparities related to healthcare, sexual and reproductive health as well as housing inequities. Conclusively, the review accentuates the need for sub-Saharan African countries to adopt low-cost preventive measures such as discouraging mass gatherings (e.g., local community gatherings), and face masking with non-medical cloth like masks for the local populace as these are crucial in managing the spread of the virus among disproportionate women population. For localities with limited access to handwashing facilities, alternative strategies like alcohol-based hand rub solutions could be deployed. The complex interrelated disparities require a broad set of policy actions to lessen the current burden faced by many women in sub-Saharan Africa.

## Introduction

Pandemics have often resulted in international, national and sub-regional crises, with considerable inequities across many societies. The novel COVID-19 disease, till date, has placed enormous burden on individuals due to stringent mitigating interventions (e.g., lockdowns, social/physical distancing measures) and causing further inequities in many vulnerable populations (e.g., low socioeconomic backgrounds) (1).

Literature on racial, gender, and social class disparities suggest that individuals from vulnerable or marginalized backgrounds often experience marginalization and discrimination across many social systems (e.g., education, health, labor) (1, 2). According to Kantamneni, challenges associated with COVID-19 could reinforce and exacerbate disparities (e.g., health, unemployment) because of limited resources (3).

With the already existing structural and socio-economic inequalities in sub-Saharan Africa, the stay-at-home orders, lockdowns, and shutdowns across the sub-regional states could worsen and have a tremendous impact on vulnerable groups such as women (3). Considering that numerous studies [e.g., (4–7)] across a variety of contexts have well-documented gender, and cultures on varied health outcomes, this mini review discusses the ways through which COVID-19 have disproportionately impacted the lives of vulnerable girls and women across sub-Saharan Africa and the strategies that can help mitigate these challenges.

### Disproportionate Areas Affected by COVID-19

Social determinants of health such as gender or sex uniquely presents inequalities or disparities along which COVID-19 may widen existing challenges in sub-Saharan Africa (8, 9). For instance, various separate reports from the pandemic suggest that young girls and women commonly report more physical and mental unhealthy period of the year despite using more preventive health care services compared to men (10). Available statistics reveal that women of diverse ethnic backgrounds (e.g., Canada, US) experience negative health conditions including asthma (10), diabetes (11), and myocardial infarctions (12). According to Kantamneni (1), these health inequalities are worsened in young girls and/or women from low socioeconomic background (SES), those with no or low education, and those in rural geographic locations. Therefore, COVID-19 pandemic could further widen existing inequalities (e.g., healthcare, income, education) within sub-Saharan African societies with serious repercussions.

#### Household Inequities

The COVID-19 pandemic has already shown unequal gender roles and household inequities across many societies (13). According to Haynes, the burden of household care has been an enormous barrier against women's socioeconomic development universally. Although parents have been affected by school closures, job losses, and primarily working from home to observe social/physical distancing measures, women, especially mothers, have been saddled with multiple responsibilities for work (i.e., formal/informal), domestic work, and childcare compared to men and/or fathers (14). Available records show that while fathers have also seen increased childcare responsibilities during the pandemic, women's childcare related tasks during lockdown have increased to more than 3 extra h per day than the reported 2 h for men averagely (15). For example, the American Time Use Survey investigated household labor divisions across gender and revealed that a higher percentage of women (84%) spent more hours (2.6 h) per day on household activities when compared to men (69%) who spent averagely (2.0 h) on domestic activities (16). The survey also showed that women spent nearly two times the period for offering child care at home than men (16). Given that patriarchal and socio-cultural norms and structural inequalities favor men, including household responsibilities in many parts of sub-Saharan Africa, the COVID-19 pandemic may place additional burden of household work and childcare on young girls and women. Although sub-regional specific data is not readily available, other customarily roles may create additional burden of elder care and care of the sick, relatives and the vulnerable during the pandemic may exacerbate the already household burden for young girls and women compared to men (17). With sub-Saharan Africa noted as one of the regions with the highest prevalence of intimate partner violence globally (18–20), these household inequalities (i.e., domestic family responsibilities) during the pandemic could create multiple role conflicts that could trigger domestic violence (e.g., physical, sexual and emotional abuse) against women, and create potential lasting mental health consequences in the region (18, 19, 21).

#### Educational Inequalities

The academic environment for learning has been acknowledged to have long-lasting implications on the educational outcomes of children (22). However, the current pandemic has caused substantial interruptions to the educational settings worldwide. Since the outbreak of the COVID-19, day care centers, schools, colleges, and universities have had to be closed by various governments as a preventive strategy to control the spread of COVID-19. These school closures have affected nearly 90% of students across all levels, with the highest proportion, over 800 million being girls and young women (23). Significantly, majority of these vulnerable girls live in least developed nations where access to education is already problematic so COVID-19 could worsen the existing inequities (24). With the already existing socio-cultural and structural barriers to girls/women's education and other limited empowerment opportunities in sub-Saharan Africa, COVID-19 may cause disproportionate drop out of school by teenage girls and young women for varied socio-economic reasons (e.g., unwanted pregnancies, manual labor). Experiences from the past Ebola outbreak show that school closures led to increases in teenage pregnancies after resumption (25). These young girls were subsequently barred from returning to school, according to Elston and colleagues. It is more likely that the current pandemic could cause similar challenges with female drop-outs in sub-Sahara Africa, where adolescent pregnancy prevalence rate is reportedly higher than other regions (26, 27). From a socio-cultural perspective, adolescent or teenage girls and women may have limited time studying than male counterparts because of increased domestic responsibilities during the pandemic (23). Socio-cultural norms in many parts of sub-Saharan Africa devalue girl child education and rather favor a boy child educational training (28, 29). Therefore, it is more likely that more teenage girls and young women could be encouraged by parents and family to choose alternate arrangements (e.g., job placement, early marriage) at the expense of their education upon school resumption. Therefore, the current COVID-19 pandemic could cause more females than males serving at home, with limited studying opportunities, and dropping out of school if necessary interventions are not implemented by various sub-regional governments and educational institutions.

#### Work/Employment Inequalities

According to the International Labor Organization, approximately 2·7 billion people, 81% of the world's working population have been seriously affected by COVID-19 related lockdown interventions (e.g., social/physical distancing protocols). Of this proportion, nearly 61% of these workers are reportedly from the informal sector, of whom 90% are in low-income and middle-income countries, including sub-Saharan Africa (30). With unemployment and low income rates already high in the sub-Saharan region (31), the intervention protocols associated with the pandemic could further widen and cause disproportionate number of employee challenges. Available evidence shows that majority of women in sub-Saharan Africa live on the fringes of the peripheral sectors of the African economy, with common economic engagements such as small-scale farming, petty trading, small enterprises, and domestic tasks with minimal financial rewards (32–34). Besides, among several groups of workers that have been deemed “essential” and required to be physically present at work are health care professionals (35). These health care workers are considered as frontline staff against COVID-19 who are not only at higher risk of infection but are also under significant psychological stress due to enormous work schedules (36, 37). COVID-19 institutional actions have inadvertently deepen the vulnerability of workers, especially among women who form the largest proportion of the nursing staff in many health facilities in the region (38–40). As health care professionals, women multiple role conflict between work schedules and family life (i.e., more household and caretaking responsibilities) during the pandemic may expose them to heightened risk and enormous pressure compared to men (1). Again, due to workplace expectations, and sociocultural norms associated with parenting and household responsibilities in sub-Saharan Africa, women are likely to experience additional strain during COVID-19 to manage and/ or balance these multiple roles. Therefore, women are likely to prioritize their increased domestic responsibilities over their professional roles and minimize professional responsibilities because of the demanding nature of undertaking both schedules (1, 16).

This perspective of gender exclusion in the region demonstrates low socio-economic welfare experience of women because of the somehow limited opportunities in the formal labor market and further mirrors the inequality burden (32). These existing inequities have been exacerbated by the pandemic which might lead to disproportionate impacts on women's well-being and their economic growth (35). Some of these marginalized women who are extremely burdened by the harsh living conditions at home (e.g., living crowded rooms, poor social amenities- housing, poor drinking water, limited electricity supply, no or poor internet access) will suffer more from the fallout (e.g., shuttering of small businesses, loss of income) of the current pandemic if some drastic interventions (e.g., setting micro-finance schemes) to alleviate their burdens are not implemented. According to some scholars [e.g., (32, 33, 41)], the increasing growth of inequities decrease the response of poverty reduction to socio-economic growth, with this gender inclusion likely to affect the achievement of the SDGs related to extreme poverty reduction across the continent. Therefore, work institutions should implement measures (e.g., provide incentives or rewards, psychological support) that keenly promote the well-being of women (35). Future empirical research is required to explore what structural barriers hinder young girls and women's access to employment during the COVID-19 pandemic. These studies could consider the short-term and long-term socioeconomic, vocational, and psychological consequences for this vulnerable group regarding access to basic needs, survival, and the disparity rates caused by COVID-19 (1).

#### Disparities Related to Healthcare, Sexual and Reproductive Health

Previous history suggests that pandemics create limited access to the healthcare, particularly with preventative and reproductive healthcare (42, 43). Experiences of previous pandemics (e.g., SARS, Ebola) and the current COVID-19 have shown increases in existing gender health inequalities in reproductive health care across many societies, with many compromised healthcare systems (42). For example, there have been reported increases in cesarean rates among SARS-CoV-2 positive patients, which also heighten the risk of maternal and neonatal complications (44, 45). With already disproportionate individual (e.g., no or low education, low income) and contextual barriers (e.g., transportation, geographical location, system organizational challenges, limited availability of healthcare services, health information, health infrastructure) in sub-Sahara Africa obstructing women's access to healthcare in the region (46, 47), the current pandemic will further worsen the existing poor obstetric and neonatal health conditions and increase rates of maternal and child morbidity as well as mortality in the region (48). It has been well-documented that gaps in access to and utilization of healthcare services, healthcare provider and institutional biases contribute to these negative outcomes during pandemics [(15, 49, 50)]. Further, the pandemic could cause inadequate suitable antenatal and postnatal care, which can seriously impact on the health of women's families, thus increasing children's probability of developing comorbidities and possible mortalities in disadvantaged settings (51, 52).

Healthcare obstacles peculiar to women are not always physical barriers in sub-Saharan Africa, but also cultural barriers (e.g., power distances, masculinity-femininity orientation) connected with gender usually discourage women from seeking access to healthcare. These cultural barriers could worsen access to healthcare during the pandemic, especially in populations where traditional practices are deeply rooted in everyday life of the people. Breaking these socio-cultural barriers require novel guidance through strategies like persuasive communication and adequate information to minimize or eliminate gendered cultural norms associated with health seeking behaviors and mainstream health services to manage the spread of the virus.

#### Housing Inequities

Majority of women in sub-Saharan Africa live in deprived areas (53, 54). Whereas, substandard and inadequate housing conditions affect the general population in Africa, women are more disadvantage than men (55). These unpleasant housing conditions have enormous effects for women (56). With these substandard and inadequate housing conditions, the burden of COVID-19 could be noticeable in these unstable housed populations often occupied by women and their children. Some scholars have reiterated that living in poor housing conditions (e.g., shelters, crowded areas, access to clean water) make social distancing measures difficult and restricts one's capacity to conform with hand washing and other hygiene protocols to prevent the virus infection and local transmission (57, 58). Therefore, women living in these areas have increased risk for severe complications from virus infection. With the current happenings in the sub-region, gender responsive planning and interventions that are safe and inclusive for women need to be provided by responsible governments (59, 60).

This mini review has some limitations. First, COVID-19 inequities may vary from country to country, hence the current circumstances surrounding the pandemic makes it difficult to gather within and between country specific trends and enormity of the challenge based on empirical data. Second, because of sparse empirical information of the theme, this write-up is restricted in scope and may lack research accuracy. Additionally, data regarding gendered related variations during Covid-19 are limited. Despite these shortcomings, this conceptualized article has research, public, and policy relevance. Future empirical work could investigate within and between country trends and magnitude of COVID-19 related inequities affecting women in sub-Saharan Africa.

## Conclusions

With limited resources across many sub-Saharan member states, adopting and complying with context-specific low-cost preventive measures such as discouraging mass gatherings (e.g., local community gatherings), and face masking with non-medical cloth as masks for the local populace would be crucial in managing the spread of the virus among disproportionate women population. Other complimentary strategies are effective health education and promotion campaigns for personal hygiene, and hand washing, including cultural barriers to healthcare. For localities with limited access to handwashing facilities, alternative strategies like alcohol-based hand rub solutions could be deployed. Mandatory use of local-made or other protective nose masks by people in public places should be encouraged. The idea is that these local productions could help reduce antimicrobial resistance and other upper respiratory tract infections in low-and middle-income countries (LMICs) often worsened by poor hygiene, and overcrowded living conditions, and lack of adequate infrastructure [see (40, 61) for details]. Women leadership groups should be seen wearing these masks in public places to serve as an example for the local populace. Outreach programmes could incorporate local community leaders to emphasize women citizenry adherence to the preventive measures against the spread of the virus. Protecting local jobs and other small enterprises for women should also be a priority. Leveraging on the current pandemic to develop infrastructural deficit and other opportunities to accommodate the existing disparities of the identified sectors of various economies by African governments will protect livelihoods.

Summarily, it is clear that the COVID-19 pandemic could exacerbate existing inequities (i.e., household, educational, employment, healthcare, housing) and requires a well-planned policy response strategies for these inequalities from worsening more. COVID-19 happenings should make public healthcare and social care drive national goals in the fight against the pandemic. Understanding the role of how COVID-19 can exacerbate these identified areas and inherent challenging experiences related to inequities is crucial if such inequalities are to be addressed appreciably. These barriers provide increased susceptibility for women in the sub-region. Overall, the somehow complex interrelated disparities also require a broad set of multi-sectoral policy actions by individual governments and other stakeholders to lessen the current burden faced by many women in sub-Saharan Africa. Empirical research to investigate gender-racial-ethnic disparities on COVID-19 outcomes in the region is needed.

## Author Contributions

JH and BA conceived the idea. JH, BA, EA, A-AS, and TS prepared the initial draft of the manuscript. All authors contributed to the article and approved the submitted version.

## Conflict of Interest

EA is affiliated to L & E Research Consult. The remaining authors declare that the research was conducted in the absence of any commercial or financial relationships that could be construed as a potential conflict of interest.

## Publisher's Note

All claims expressed in this article are solely those of the authors and do not necessarily represent those of their affiliated organizations, or those of the publisher, the editors and the reviewers. Any product that may be evaluated in this article, or claim that may be made by its manufacturer, is not guaranteed or endorsed by the publisher.
